# Dissociative Symptoms in Bipolar Disorder: Impact on Clinical Course and Treatment Response

**DOI:** 10.3389/fpsyt.2021.732843

**Published:** 2021-10-25

**Authors:** Luca Steardo, Elvira Anna Carbone, Enrica Ventura, Renato de Filippis, Mario Luciano, Cristina Segura-Garcia, Pasquale De Fazio

**Affiliations:** ^1^Psychiatric Unit, Department of Health Sciences, University Magna Graecia of Catanzaro, Catanzaro, Italy; ^2^Psychiatric Unit, Department of Medical and Surgical Sciences, University Magna Graecia of Catanzaro, Catanzaro, Italy; ^3^Department of Psychiatry, University of Campania “L. Vanvitelli”, Naples, Italy

**Keywords:** dissociative symptoms, bipolar disorder, clinical course, treatment response, psychotic symptoms

## Abstract

**Background:** Dissociative symptoms are under recognized and scarcely studied by clinicians and researchers in patients with bipolar disorder (BD). We examined the relationship between dissociative symptoms and the psychotic features in patients with BD and assessed clinical and socio-demographic characteristics more frequently associated with dissociative symptoms and treatment response.

**Methods:** Participants were 100 adult outpatients with BD. They were screened with semi-structured interview to collect socio-demographic and clinical characteristics; the Dissociative Experiences Scale-II (DES-II) and the ALDA scale were used to assess dissociative psychopathologies and response to treatment with mood stabilizers, respectively.

**Results:** DES score (mean 31.7 ± 21.7) correlated with clinical variables, BD features, and course of illness. Psychotic symptoms, mixed features, and previous suicide attempts significantly predicted DES score [*F*_(3, 47)_ = 39.880, *p* < 0.001, *R*^2^ corrected = 0.713]. Dissociative symptoms were inversely correlated with poor response to treatment (*r* = −0.593; *p* < 0.001).

**Limitations:** Cross-sectional design with a small sample and backward clinical assessment of psychotic symptoms.

**Conclusions:** Dissociative phenomena are closely related to the presence of psychotic symptoms, mixed features, and previous suicide attempts in BD, especially in BD-I. Given the close association between dissociative and psychotic symptoms, this association could represent a diagnostic indicator of BD-I that may guide the clinician to plan the most appropriate treatment.

## Introduction

Dissociative symptomatology includes a variety of processes and phenomena along a continuum from adaptive coping strategies to more pathological states (1). The 5th edition of the Diagnostic and Statistical Manual of Mental Disorders (DSM-5) defines dissociative symptoms as “a disruption of and/or discontinuity in the normal integration of consciousness, memory, identity, emotion, perception, body representation, motor control, and behavior,” and identifies five components of dissociation: depersonalization, derealization, amnesia, identity confusion, and identity alteration (2).

Two major theories have been proposed to explain the etiology of dissociative symptoms. The “*trauma theory”* considers dissociation as the coping strategy adopted to deal with severe anxiety symptoms associated with traumatic experiences (3–5). On the other hand, the “*disrupted sleep theory”* considers the distortion of cognitions, emotions, and sensations associated with prolonged sleep deprivation as the trigger for the experience of dissociative symptoms (6, 7).

Current evidence suggests that the assessment of dissociative symptoms could be useful in differentiating patients with BD from those with unipolar major depression during depressive episodes. It has been reported that patients with BD are more likely to report dissociative symptoms than those patients with unipolar depression (8). Moreover, dissociative symptoms may precede the onset of the disorder and could be a risk factor for the development of BD (9). Besides, dissociative symptoms are frequently associated with a worse prognosis and a greater number of mood fluctuations (10–14). Further, available data suggest that the presence of dissociative symptoms is considered a marker of poor treatment response in BD (15). Despite dissociative symptoms can have a potential impact on the psychopathological burden of patients with BD, only a few studies have assessed the relationship between dissociative and psychotic symptoms and treatment response to mood stabilizers.

The aims of the present study are to assess: (1) the relationship between the presence of dissociative symptoms and psychotic symptoms in patients with BD; (2) the clinical and socio-demographic characteristics more frequently associated with dissociative symptoms; and (3) the potential role of dissociative symptoms on the response to treatment with mood stabilizers.

## Methods

### Participants and Procedures

This is a naturalistic and uncontrolled cross-sectional observational study. Patients that consecutively referred to the psychiatric outpatients unit of the University Hospital Mater Domini of Catanzaro, from May 2019 to January 2020 were recruited. Trained psychiatrists evaluated every participant. Patients underwent a clinical interview by means of the Structured Clinical Interview for DSM-5 (SCID-5_CV) (16) to make the diagnosis and to ascertain if participants were eligible according to all the inclusion and exclusion criteria.

Patients were included in the study if they met the following criteria: (1) age between 18 and 65 years, (2) diagnosis of type-I (BD-I) or type-II (BD-II) bipolar disorder according to the Diagnostic and Statistical Manual of Mental Disorders-fifth edition (DSM-5) (2) clinically stabilized. Patients were deemed stabilized if item 1 (severity of the disease) of the Clinical Global Impressions for Bipolar Patients (CGI-BP) scale was ≤ 2 (17). No other inclusion criteria were selected to obtain a sample as similar as possible to patients routinely seen in the real world. Patients were considered ineligible in case of: clinically diagnosed dementia, diagnosis of intellectual disability from mild to severe according to DSM-5 (corresponding to IQ < 70); alcohol or substance abuse over the last twelve months; another medical condition associated with psychiatric symptoms (e.g., temporal lobe epilepsy, multiple sclerosis, brain trauma, malignant disease); or conditions that did not allow the completion of the assessment (eg. language problems, dyslexia, or poor knowledge of the Italian language).

After receiving a full description of the study aims, all participants provided written informed consent according to the Ethical Committee's guidelines. The study was carried out in accordance with the latest version of the Declaration of Helsinki and was approved by the local Research Ethics Committee of “Regione Calabria, sezione Area Centro” (N. 307).

A statistical power analysis was done with Gpower 3.1 for sample size estimation; with an alpha = 0.05 and a power = 0.95, the projected sample size needed with an effect size = 0.15 was minimum 89 participants.

### Assessments

#### Socio-Demographic and Clinical Characteristics

Recruited patients underwent a semi-structured interview to collect socio-demographic and clinical characteristics using an *ad-hoc* schedule. Data on age, gender, civil status, education, occupation, family history of psychiatric illnesses, medical or psychiatric comorbidity, onset and longitudinal course of the disorder (number of depressive/hypo/manic episodes), suicidal ideation, and previous psychiatric hospitalizations and ongoing treatment were recorded.

#### Dissociative Experiences Scale-II (DES-II)

DES-II is a self-assessment measure developed by Bernstein and Putnam (18) and translated into Italian for the screening of dissociative psychopathologies. It consists of 28 items that describe dissociative experiences (amnesia, absorption, depersonalization, and derealisation). Patients are asked to indicate with a percentage the frequency they have experienced them (0% never; 100% always). The final score is given by the sum of the scores of the individual items divided by the number of total items and can therefore be included between 0 and 100: scores >30 are associated with a diagnosis of dissociative disorder.

#### ALDA Scale

The ALDA rating scale assesses response to treatment with mood stabilizers in bipolar patients (19–21). The scale comprises two sections to rate lithium response Section A Section B, and a total score. Section A evaluates the effectiveness of the ongoing treatment with a mood stabilizer, with a score ranging from 1 to 10 (1 = no response; 10 = excellent response); Section B consists of 5 items to be scored from 0 to 2 to be subtracted from criterion A. The five subdomains of criterion B consider the number of episodes before the start of treatment, the frequency of episodes outside the treatment period, the duration of treatment, compliance with therapy, and the presence of polypharmacy. The total score on the Alda scale is divided into three sub-items: 0–3 = poor response; 4–6 = moderate response; 7–10 = good response.

### Statistical Analyses

Statistical analyses were performed with the Statistical Package for Social Sciences, version 26 (SPSS, Chicago, IL,USA). Descriptive statistics were calculated for socio-demographic and clinical characteristics as well as for other relevant assessment instruments. Data are presented as means and standard deviations (SD) or frequencies and percentages (%), as appropriate. The association between DES and the most relevant clinical characteristics was studied through Spearman's correlation test. Forward stepwise multiple linear regression analysis was run to ascertain the independent predictors of DES-II score among clinical variables and BD features controlling for sex and age. Finally, the association between DES and Alda scores was studied with Pearson's correlation analysis.

The level of statistical significance was set at *p* < 0.05.

## Results

The total sample consisted of 100 patients (55 BD-I and 45 BD-II), 50% males, mean age 46.5 ± 13.9 years. Table 1 illustrates the socio-demographic and clinical characteristics of participants according to their diagnosis. Almost half of patients were in a stable relationship, employed and had a positive history for psychiatric disorders. The mean age at onset was 27.30 ± 9.85 and the duration of illness was 19 ± 12.99 years. Significant differences emerged in clinical variables, BD features and ALDA score according to diagnosis. Only patients with BD-I reported psychotic symptoms (43/55); patients reported psychotic symptoms during manic (51%), depressive (9%), or both episodes (40%).

**Table 1:** Socio-demographic and clinical characteristic of total sample.

			**DB-I**		**DB-II**		**Comparison**	
			***N*** **= 55**		***N*** **= 45**			
			**Mean**	**SD**	**Mean**	**SD**	**Statistics**	***P***
Socio-demographic characteristics	Age		45.0	14.4	48.3	13.3	*t* = −1.178	0.242
	Gender	Male	27	54	23	46	χ^2^ = 0.040	0.841
	Education	Years	13.3	3.6	13.8	3.0	*t* = −0.684	0.495
	Having partner^*^	Yes	45	82	39	87	χ^2^ = 0.433	0.511
	Employed^*^	Yes	30	55	30	67	χ^2^ = 1.515	0.218
	Family history of psychiatric disorders^*^	Yes	39	57	29	43	χ^2^ = 0.475	0.491
Clinical variables	Age at onset		24.5	5.4	30.7	12.7	*t* = −3.307	**0.001**
	Age first psychiatric contact		27.6	6.3	32.9	12.8	*t =* −2.730	**0.008**
	Age first depressive episode		29.2	11.0	25.2	8.9	*t =* 1.942	0.055
	Age first maniac episode		28.6	6.9	27.6	7.4	*t =* 0.504	0.617
	Age first hypomaniac episode		33.4	10.9	29.3	8.4	*t =* 1.895	0.062
	Tot number of depressive episodes		7.5	5.9	2.9	1.4	*t* = 4.961	**<0.001**
	Tot number of manic episodes		3.8	2.9	0	0		
	Tot number of hypomanic episodes		3.8	3.9	2.6	1.1	*t* = 2.002	**<0.05**
	Tot number of episodes		14.7	11.5	5.4	2.2	*t* = 5.319	**<0.001**
	Tot number episodes last year		0.8	0.8	0.8	0.7	*t* = −0.092	0.927
	Suicide attempts^*^	Yes	28	88	4	13	χ^2^ = 20,083	**<0.001**
	Aggressive behaviors^*^	Yes	41	72	16	28	χ^2^ = 15.351	**<0.001**
	Psychotic symptoms^*^	Yes	43	78	0	0		
	Seasonality^*^	Yes	21	41	25	57	χ^2^ = 2.314	0.128
	Tot number of hospitalizations		0.7	0.9	0.6	0.9	*t* = 0.533	0.596
	Illness duration	Years	20.5	14.2	17.6	11.1	*t* = 1.138	0.258
	Course of illness^*^	Irregular	16	29	16	36	χ^2^ = 0.475	0.491
BD features	Anxious features^*^	Yes	44	70	19	30	χ^2^ = 15.153	**<0.001**
	Mixed features^*^	Yes	37	77	11	23	χ^2^ = 19.090	**<0.001**
	Antidepressants switch^*^	Yes	24	83	5	17	χ^2^ = 12.716	**<0.001**
Current treatment	Antipsychotics^*^	Yes	47	85	36	80	χ^2^ = 0.522	0.47
	Antidepressants^*^	Yes	8	15	5	11	χ^2^ = 0.258	0.611
	Lithium^*^	Yes	32	58	27	60	χ^2^ = 0.034	0.854
	Mood stabilizers^*^	Yes	31	57	27	60	χ^2^ = 0.134	0.714
	Combined therapy^*^	Yes	47	85	39	87	χ^2^ = 0.030	0.862
ALDA	Total score		3.6	2.0	4.8	2.0	*t* = −3.022	**0.003**
	Response^*^	Poor	29	52	11	24	χ^2^ = 10.286	**0.006**
		Moderate	23	42	25	56		
		Good	3	6	9	20		
DES-II	Total score		43.0	19.4	18.0	15.6	*t* = 6.963	**<0.001**

* *Data are presented as frequencies and percentages (%). Significant results are in bold*.

*BD-I, type I bipolar disorder; BD-II, type II bipolar disorder; DES II, Dissociative Experience Scale II*.

Table 2 includes the results of Spearman's correlations between DES-II and the clinical features of patients. Significant correlations emerge for all variables except age at onset of bipolar disorder. Successively a linear regression analysis was performed to determine the relationship between clinical variables and DES. Successively a linear regression analysis was performed to determine the relationship between clinical variables and DES. The results indicated that there was a significant effect of clinical variables on DES [*F*_(3, 47)_ = 39.880, *p* < 0.001, *R*^2^ corrected = 0.713). The individual predictors were examined further and indicated that mixed features (*t* = 3.762, *p* < 0.001), psychotic symptoms (*t* = 4.932, *p* < 0.001 and previous suicide attempt (*t* = 3.928, *p* < 0.001) were significant predictors in the model (Table 3).

**Table 2:** Spearman's correlations between DES-II and clinical features.

	**DES score**
Tot number of episodes	0.533^**^
Antidepressants' switch	0.497^**^
Suicide attempts	0.551^**^
Psychotic features	0.759^**^
Aggressive behavior	0.339^**^
Mixed features	0.674^**^
Anxious features	0.603^**^
Course of illness (regular)	−0.417^**^
Seasonality	0.368^**^
Age at onset	−0.063

** *p < 0.01. Significant results are in bold*.

**Table 3:** Multiple linear regression model.

**Dependent variable**	**Independent variable**	**B**	**Standard error**	**Beta**	***t***	***P***	**95% Confidence interval**	
							**Lower**	**Higher**
DES-II	Mixed features	13,964	3,711	0,359	3,762	< .001	6,484	21,443
	Psychotic features	16,23	3,291	0,444	4,932	< .001	9,598	22,862
	Previous suicide attempt	11,673	2,972	0,33	3,928	< .001	5,684	17,663

Finally, a significant negative association between DES and ALDA scores (*t* = −0.593; *p* < 0.001) emerged from the correlation analysis.

## Discussion

The main finding of our study is the presence of a stable correlation between dissociative symptoms psychotic symptom, mixed features, and previous suicides attempts in BD. Our study is the first to demonstrate that the occurrence of dissociative dimension is higher in BD type I, especially in those with psychotic features and in those who present a greater clinical severity (22–24). In our sample, 45% of patients developed psychotic symptoms, all of them with BD-I. Moreover, such correlation is related to clinical severity as shown by the higher DES-II total score in patients with a higher number of episodes, aggressive behaviors, seasonality, mixed features, and poor response to mood stabilizers (25). Possibly, as previously demonstrated for other mental disorders, dissociative symptoms are more expressed in patients with psychotic symptoms (26). According to Allen et al. (27), who developed the theory of “dissociative detachment,” dissociative phenomena “undermines the individual's grounding in the outer world,” rendering the individual susceptible to developing psychotic features. Additionally, other authors have highlighted the inverse relationship in which dissociation is a defense against the disorganization and acute distress generated by psychosis (28). Other possible mechanisms might be the early trauma exposure that predisposes to a higher psychopathological load among different psychiatric disorders, including BD (29, 30). However, we did not assess the impact of trauma in this study and therefore this explanation remains speculative.

In addition, mixed characteristics were associated with a higher DES-II total score. Mixed features are associated with a lack of response to mood stabilizers in BD and this could explain why patients with dissociative are refractory to treatment (31). Mixed features are often triggered by antidepressant therapy, and this feature appeared associated with greater dissociative symptomatology as demonstrated in our study.

Patients with a lifetime history of suicide attempts reported higher rates of dissociative symptoms and a higher DES-II total score. Suicide could represent an attempt to cope with dissociative symptoms or a last attempt to reach a dissociative state (32, 33). Thus, the link between dissociative symptoms and suicidal behaviors represent an interesting topic in order to improve the risk assessment and to develop models of intervention in patients with BD.

The significant correlation between DES and poor response to treatment with mood stabilizers emerged. We can explain it because clinical variables associated with dissociative symptoms are superimposable with those that predispose to refractoriness to drug treatment. In fact, this correlation gains further support from the significant correlations between clinical variables and dissociative symptoms that emerged from our study (i.e., the higher number of lifetime episodes, the seasonality, the antidepressant switch, the aggressive behavior, the anxious features, and the irregular course of illness) that are all predictive factors of poor response to treatment for patients with BD. So of poor response to treatment in patients with bipolar disorder (34).

The authors acknowledge some limits, the first being the cross-sectional design with a small sample size that narrows conclusions about the reverse causality. Surely, a longitudinal evaluation of dissociative symptoms would allow clarifying their association with clinical variables. Another limit is the backward clinical assessment of psychotic symptoms. The recall bias of incompleteness and imprecision of the patients' memories represents another limitation. Ultimately, patients completed the protocol while clinically stabilized so, assessing dissociative symptoms during the acute phase of the BD, although interesting was not possible. For all these reasons, the authors have planned follow-up assessments and including patients in the acute depressive, manic, or hypomanic episodes to counterbalance these limits.

## Conclusions

Dissociative phenomena are closely related to the presence of psychotic symptoms, mixed features, and previous suicide attempts in BD, especially in BD-I. Given the close association between dissociative and psychotic symptoms, this association could represent a diagnostic indicator of BD-I. Therefore, we consider it extremely important to evaluate the presence of ongoing dissociation in BD to guide the clinician to plan the most appropriate treatment. Future studies should investigate in greater depth the causes underlying this association and the most appropriate therapeutic strategies to reduce the impact that these symptoms have on the course of BD.

## Data Availability Statement

The raw data supporting the conclusions of this article will be made available by the authors, without undue reservation.

## Ethics Statement

The studies involving human participants were reviewed and approved by Regione Calabria, sezione Area Centro. The patients/participants provided their written informed consent to participate in this study.

## Author Contributions

LS: conceptualization. LS and ML: methodology. LS, ML, and CS-G: formal analysis. LS, EAC, and RF: investigation. EV and RF: data curation. LS, EAC, and ML: writing the original draft preparation. CS-G and PD: writing and review and editing. All authors read and approved the final manuscript.

## Conflict of Interest

The authors declare that the research was conducted in the absence of any commercial or financial relationships that could be construed as a potential conflict of interest.

## Publisher's Note

All claims expressed in this article are solely those of the authors and do not necessarily represent those of their affiliated organizations, or those of the publisher, the editors and the reviewers. Any product that may be evaluated in this article, or claim that may be made by its manufacturer, is not guaranteed or endorsed by the publisher.

## Abbreviations

BD: Bipolar Disorder
BD-I: Bipolar Disorder type I
BD-II: Bipolar Disorder type II
CGI-BP: Clinical Global Impressions for Bipolar Patients
DES-II: Dissociative Experiences Scale-II
DSM-5: Diagnostic and Statistical Manual of Mental Disorders 5th edition.
